# How to translate genetic findings into clinical applications in spondyloarthritis?

**DOI:** 10.3389/fimmu.2024.1301735

**Published:** 2024-01-24

**Authors:** Eva Frison, Maxime Breban, Félicie Costantino

**Affiliations:** ^1^ UMR1173, INSERM, UFR Simone Veil, Versailles-Saint-Quentin-Paris-Saclay University, Saint-Quentin-en-Yvelines, France; ^2^ Labex Inflamex, Paris Diderot Sorbonne Paris-Cité University, Paris, France; ^3^ Rheumatology Division, Ambroise Paré Hospital, Assistance Publique des Hôpitaux de Paris (AP-HP), Boulogne-Billancourt, France

**Keywords:** genetics, genomics, translational genetics, spondyloarthritis, ankylosing spondylitis, diagnosis, prognosis, therapeutic target

## Abstract

Spondyloarthritis (SpA) is characterized by a strong genetic predisposition evidenced by the identification of up to 50 susceptibility loci, in addition to HLA-B27, the major genetic factor associated with the disease. These loci have not only deepened our understanding of disease pathogenesis but also offer the potential to improve disease management. Diagnostic delay is a major issue in SpA. HLA-B27 testing is widely used as diagnostic biomarker in SpA but its predictive value is limited. Several attempts have been made to develop more sophisticated polygenic risk score (PRS). However, these scores currently offer very little improvement as compared to HLA-B27 and are still difficult to implement in clinical routine. Genetics might also help to predict disease outcome including treatment response. Several genetic variants have been reported to be associated with radiographic damage or with poor response to TNF blockers, unfortunately with lack of coherence across studies. Large-scale studies should be conducted to obtain more robust findings. Genetic and genomic evidence in complex diseases can be further used to support the identification of new drug targets and to repurpose existing drugs. Although not fully driven by genetics, development of IL-17 blockers has been facilitated by the discovery of the association between *IL23R* variants and SpA. Development of recent approaches combining GWAS findings with functional genomics will help to prioritize new drug targets in the future. Although very promising, translational genetics in SpA remains challenging and will require a multidisciplinary approach that integrates genetics, genomics, immunology, and clinical research.

## 1 Introduction

**Figure 1:**
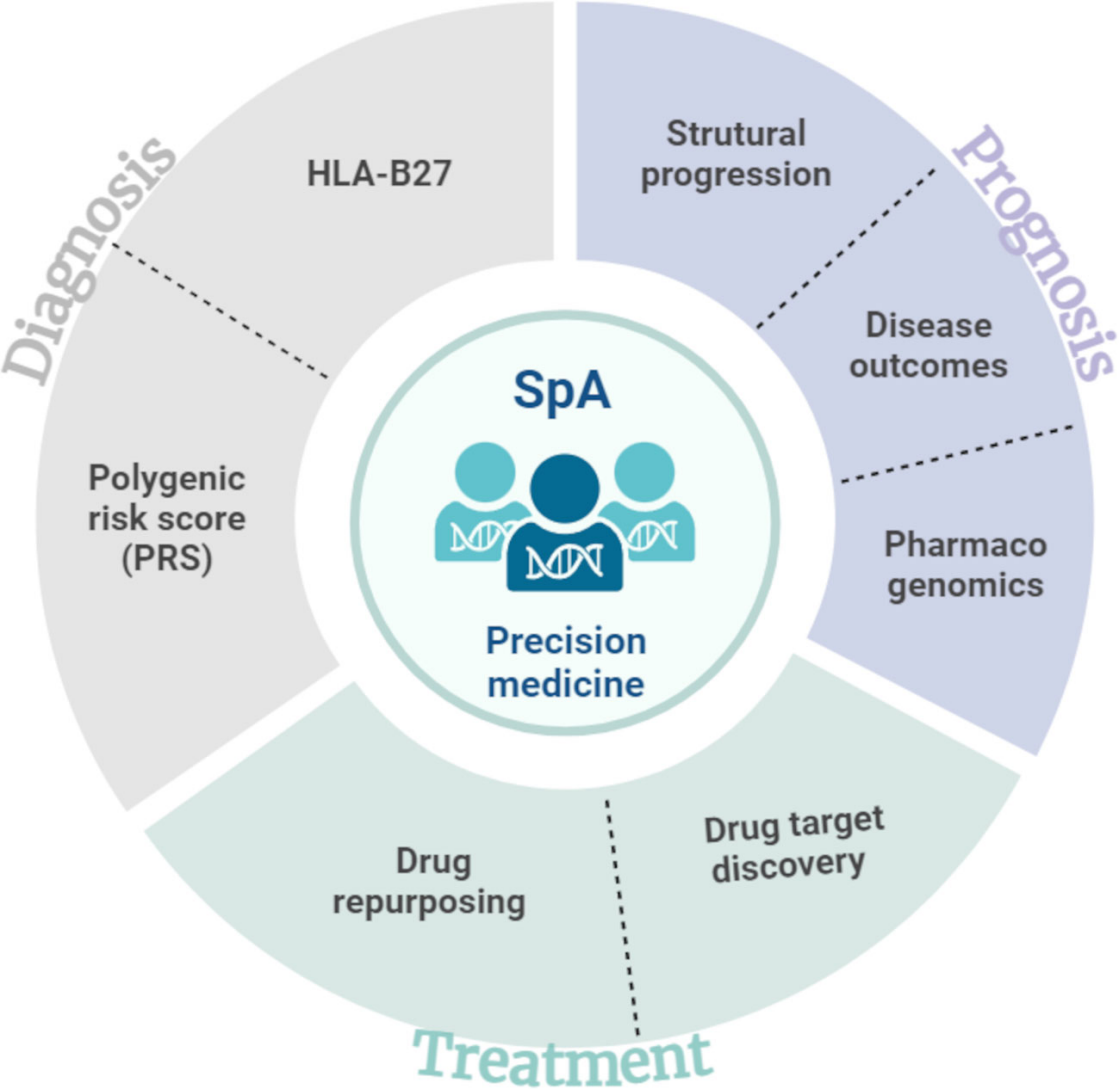
Overview of translational applications of genetic findings in spondyloarthritis.

Spondyloarthritis (SpA) is a chronic immune mediated inflammatory disease characterized by a combination of articular and extraarticular inflammatory manifestations. One of the hallmarks of this disease is its strong genetic predisposition. Following the discovery of the strong association of HLA-B27 allele with the disease, up to 50 other susceptibility *loci* have been reported through genome-wide association studies (GWAS). All these loci have provided new insights into disease pathogenesis but might also help to improve disease diagnosis, to detect patient at risk of poor outcome and to identify genetic predictors of treatment response and new drug targets. However, translating genetic findings into clinically meaningful applications remains challenging in complex diseases such as SpA. The purpose of this review is to describe the current status and outline the potential usefulness of clinical applications of genetic knowledge in SpA (**Figure 1**).

## 2 Diagnosis

Diagnosis delay is still a major issue in axial spondyloarthritis (axSpA) with an average time between symptom onset and definite diagnosis of around 5 years (1). Low specificity of most of the frequent clinical manifestations and lack of accurate diagnostic biomarkers contribute to this delay. Given the significant genetic predisposition to axSpA, use of genetic biomarkers to reduce diagnosis delay appears attractive. However, apart for HLA-B27, no single genetic marker has demonstrated noteworthy diagnostic efficacy, prompting the development of more sophisticated polygenic risk score.

### 2.1 HLA-B27

HLA-B27 testing has been widely used as diagnostic biomarker in axSpA since the discovery of its strong association with the disease (2). However, several factors limit its diagnostic performance (3). First, a substantial proportion (up to 30%) of patients with axSpA do not carry the HLA-B27 allele, affecting the sensitivity. Second, HLA-B27 allele frequency in the general population (e.g. 5 to 10% in Caucasians) is generally higher than required to offer valuable specificity (4). Diagnostic utility of HLA-B27 is also highly dependent on the pre-test probability based on clinical judgement of the physician. Indeed, to provide an acceptable post-test probability of 95%, the physician confidence on diagnosis before HLA-B27 testing should be higher than 50% (5).

Thus, HLA-B27 cannot be used alone for diagnostic purpose and needs to be combined with clinical and imaging features. Rudwaleit et al. estimated in 2004 the utility of several SpA features in axSpA diagnosis. They showed that the presence of two to three SpA features was necessary to increase the diagnostic probability of axSpA to 90% in patients with chronic back pain. With a positive likelihood ratio (LR+) of 9.0, HLA-B27 was the most useful feature together with positive sacroiliac joint MRI (LR+ = 9.0) (6).

### 2.2 Other single genetic markers

Thanks to genomewide association studies (GWAS), more than 50 independent *loci* have been associated with ankylosing spondylitis (AS) (7). Although these loci have been significantly associated with AS, the individual risk conferred by each of them is low with odds ratios ranging from 1.1 to 1.7, as compared to an odds ratio of 46 for HLA-B27 (8). Therefore, diagnostic value of single variant is negligible and has no interest in daily practice.

### 2.3 Polygenic risk scores

Instead of focusing on a single genetic marker to assess genetic risk, an increasingly popular approach is to use aggregate measures of several genetic risk factors into polygenic risk scores (PRS). The initial strategy was to use only *loci* significantly associated with the disease to elaborate PRS (9). More recently, is has been demonstrated that models incorporating a large number of SNPs without individually significant effect outperformed those employing only GWAS-associated SNPs (10). This aligns with the evidence that a significant fraction of the heritability of complex traits relies on a large number of low-level effect polymorphisms (11).

In SpA, several attempts have been made to elaborate an accurate PRS for diagnostic purpose. In 2017, Thomas et al. demonstrated good diagnostic performance for both AS and non-radiographic axial SpA of a PRS based on the combination of 31 GWAS-associated SNPs and HLA-B27 allele (area under the curve (AUC) of 0.9 and 0.84 respectively) (12). However, the authors did not provide the AUC of using HLA-B27 alone to estimate the additional value of their PRS. A similar approach was used with more recent GWAS results by Rostami et al. (13) who developed a PRS based on 110 GWAS-associated SNPs. They showed low discrimination capacity of PRS alone (AUC = 0.62) lower than that of HLA-B27 alone (AUC = 0.88). Combination of PRS and HLA-B27 improved diagnostic prediction (AUC = 0.9) but the improvement was small and of uncertain clinical value. More recently, Li and al. developed two PRSs based on a larger number of SNPs in two distinct ethnic populations (European descent and East-Asian). These PRSs have good predictive performance in AS, slightly outperforming HLA-B27 alone or MRI (14). Moreover, they showed that PRSs performances were better if developed in the ethnic group to which they are to be applied.

Recent work suggests that rare variants that are poorly tagged by common variants can explain part of missing heritability (15). Incorporating those rare variants into PRS (either as rare-variant polygenic risk score (RVPRS) or in combination with common variant risk score) might increase diagnostic performance (16).

While PRS shows promise in improving SpA diagnosis, widespread use of such score will require further research and validation. Cost-effectiveness of this kind of approach also needs to be demonstrated.

## 3 Prognosis

Identification of genetic predictors of poor prognosis could significantly improve treatment strategies in SpA. However, defining severity in SpA is challenging because it encompasses multiple domains, including pain, disease activity, physical function, radiographic structural damage, and treatment response. Disease severity is at least partially genetically determined as demonstrated by high heritability of disease activity functional impairment and radiographic damage (17, 18). Until now, a majority of research efforts have been focused on the identification of genetic predictors of radiographic progression.

### 3.1 Radiographic severity

Studying genetic factors associated with radiographic damage in SpA raises significant challenges. First, it requires available spinal X-rays of good quality to allow the calculation of a reliable scoring system such as mSASSS (modified Stoke Ankylosing Spondylitis Spine Score) (19). Studied population also needs to be well characterized, including information on factors known to be associated with radiographic progression such as male sex, older age, longer disease duration, elevated CRP, smoking status, CRP level or TNF blockers use (20).

Genetic studies targeting radiographic damage in axSpA are still sparse. Most of the studies focused on SNPs in well-established AS susceptibility *loci*, such as *ERAP1* (Endoplasmic Reticulum AminoPeptidase 1), *IL23R* (Interleukin 23 Receptor) or HLA region or on candidate genes involved in ossification or bone remodelling. Those studies reported associations with several genes, including HLA-B27 (21), *RANK* (Receptor Activator of Nuclear factor Kappa B) and *PTGS1* (Prostaglandin-Endoperoxide Synthase 1) (22), *FGB* (FibrinoGen Beta chain) (23), *LMP2* (Latent Membrane Protein 2) (24), *ADRB1* (ADRenoceptor Beta 1), *NELL1* (Neural EGFL Like 1) (25), *IL23R* (26) and *TAP2* (Transporter 2, ATP binding cassette subfamily B member) (25). However, most of them did not reach statistical significance and none of them was independently replicated. More recently, Nam et al. performed in a Korean population the first GWAS focused on radiographic severity (27). The best associated SNP in this GWAS was an exonic variant in *RYR3* (RYanodine Receptor) but it did not reach genomewide significance threshold. However, given the sample size of the study (444 AS patients), the classical GWAS significance threshold of 5x10^-8^ might be too conservative and authors provided functional data linking RYR3 with matrix mineralisation which is consistent with a possible role in structural damage in AS.

### 3.2 Other disease outcomes

Genetic studies have also been conducted on other disease outcomes such as BASDAI or BASFI. However, all those studies suffered from a limited statistical power and a lack of consistent replication, thereby preventing definite conclusions from being drawn, except for the HLA-B27 allele. Indeed, current evidence suggests that B27-positive patients with axSpA might suffer from a worse disease prognosis than B27-negative patients, with higher disease activity (as measured by CRP and ASDAS) and more frequent MRI inflammation of the SI joints and the spine (28, 29).

## 4 Treatment

### 4.1 Treatment response prediction

With the growing number of therapeutic options in axSpA, it is increasingly important to be able to predict the likelihood of treatment response and to identify the best therapeutic target for a given patient. Pharmacogenomics is an emerging field aiming at identifying genetic markers that can predict at individual level the response to a particular medication. Objectives are not only to increase treatment efficacy but also to reduce the risk of adverse drug reaction.

The genetic contribution to drug response in SpA has been recently the subject of a systematic literature review by Ortolan et al. (30). Only 26 studies of 393 screened studies were analysed after selection process, 21 of them investigating TNF blockers efficacy. The most frequent reported associations were with polymorphisms in *TNFRSF1A/1B* (TNF receptor superfamily member 1A/1B) and *TNF* but results were often conflicting. HLA-B27, not included in this systematic literature review, has also been identified as a predictor of good response to TNF blockers (31).

Implementing pharmacogenomics results into clinical practice faces many challenges, including the determination of which gene-drug pairs are actionable. The Clinical Pharmacogenetics Implementation Consortium (CPIC) has established a gene/drug database to help the identification of situations in which a given genotype results in dose modification or in drug substitution (https://cpicpgx.org/genes-drugs/). Interrogation of this database shows limited evidence for actionable gene/drug pairs in the field of SpA (**Table 1**). In fact, high level of evidence of actionable gene/drug pairs was only found for non steroidal anti inflammatory drugs and *CYP2C9* (cytochrome P450 family 2 subfamily C member 9) (32).

**Table 1:** Summary of CPIC levels for genes/drugs pairs and recommendations for drugs used in SpA.

Drug class	Gene	Drug	CPIC Level*	Recommendation
NSAIDS	*CYP2C9*	Piroxicam Tenoxicam	A	In poor or intermediate metabolizers, consider alternative therapies not primarily metabolized by CYP2C9
		Meloxicam	A	In intermediate metabolizers, initiate therapy with 50% of the lowest recommended starting dose. In poor metabolizers: consider alternative therapies not primarily metabolized by CYP2C9
		Celecoxib Flurbiprofen Ibuprofen Lornoxicam	A	In poor metabolizers, initiate therapy with 25–50% of the lowest recommended starting dose or consider alternative therapies not primarily metabolized by CYP2C9
		Aceclofenac Diclofenac Indomethacin Lumiracoxib Naproxen	C	None
	*CYP2C8*	Diclofenac	C	None
		Ibuprofen	C	None
csDMARDs	*ABCB1*	Methotrexate	C	None
	*MTHFR*		C	None
	*SLCO1B1*		C	None
	*ATIC*		D	None
	*MTRR*		D	None
	*NAT2*	Sulfasalazine	B/C	None
	*G6PD*		C	None
bDMARDs	*TNF*	Adalimumab	C	None
		Etanercept	C	None
		Infliximab	C	None

NSAIDs, non steroidal anti-inflammatory drugs; csDMARDs, conventional synthetic disease modifying antirheumatic drugs; bDMARDs, biological disease modifying antirheumatic drugs.

*CPIC level: A and B: moderate to high level of evidence with prescribing action recommended; C and D: low level of evidence with no prescribing action recommended.

### 4.2 Drug repurposing or drug target discovery

New drug development is a highly expensive process with a high rate of failure. Genomics might help to decrease this failure rate as drug targets with genetic support are more than twice as likely to be successful in clinical development (33, 34). Recent development of evolocumab and alirocumab based on the discovery of *PCSK9* (Proprotein Convertase Subtilisin/Kexin type 9) mutations causing hypercholesterolemia corroborates the utility of genomics-driven drug development (35). Genomics might also be very useful for drug repurposing, a strategy in which new indications are identified for existing therapies (36). This approach has a greater likelihood of success and requires significantly less time and monetary investment. In recent years, an increasing number of methods using genetic data for systematically prioritizing drug targets have been proposed (37).

Although no genetic findings have completely driven drug development in SpA, association between AS and several variants in genes involved in IL-17/IL-23 axis have facilitated the development of secukinumab, an IL-17 blocker (38). Several studies have tried to identify candidate drugs in AS by using GWAS findings. In 2016, Ellinghaus et al. performed a cross-phenotype GWAS of 5 immune-mediated diseases including AS (7). Through integration of their GWAS findings with protein-protein interaction networks they found interesting therapeutic targets, some of them currently being tested in AS. More recently, Brown et al. used a genetics-led approach that annotates GWASs with functional genomic data to prioritize new therapeutic targets in AS (39). They found that their algorithm had good performance to prioritize currently approved drug targets for AS with some known AS drug targets such as *IL23R*, *JAK2* (JAnus Kinase 2) among the top 1% of prioritized genes. They also identified new pathways and potential drug targets, including *PTGER4* (ProsTaGlandin E Receptor 4), *ERBB* (ErbB), *PI3K* (PhosphatidylInositol 3-Kinase), *NOTCH1* (NOTCH receptor 1) and *GPCR* (G Protein Coupled Receptor).

## 5 Discussion

### 5.1 Challenges in genomics-driven precision medicine in SpA

As outlined in this review, advances in technology and decreasing costs of genetic sequencing are enhancing the feasibility of integrating genetic information into daily practice. This might help to address unmet needs in SpA, by enabling earlier diagnosis, personalized treatment plan and new drugs development. However, there are several hurdles that need to be surmounted to unlock this potential.

First, the genetic architecture of SpA needs to be better understood, with only a small fraction of disease heritability currently explained. Sample sizes of GWAS performed to date are relatively modest in comparison with other immune mediated diseases such as inflammatory bowel diseases or rheumatoid arthritis. Thus, there is a need for new GWAS with larger sample size and better coverage of the whole genome. Another critical point, especially in the perspective of drug target discovery, is the identification of causal variants and consequently of the functional mechanisms behind the genetic associations.

Moreover, diagnostic, prognostic or theragnostic biomarkers require robust validation to be usable in daily routine. This will require large scale collaborative efforts to constitute large DNA cohort of well phenotyped patients, in diverse populations, ensuring that the identified biomarkers are relevant for different ethnic groups and disease subtypes.

Integration of other “omics” technologies with genetics is also a critical step to improve precision medicine. In particular, epigenetics plays a crucial role in incorporating environmental factors into precision medicine. Analysing epigenetic modifications alongside genetic and transcriptomic data might help to understand how environmental factors contribute to disease susceptibility and progression. As epigenetic modifications are reversible, they might also be accessible to therapeutic interventions. This opens us new opportunities for tailored treatment considering both individual’s genetic background and environment.

### 5.2 Ethical and social concerns

Beyond technical and methodological challenges already described, personalized medicine also raises ethical and social issues. In a recent position paper, the American College of Physicians recommended that, before conducting any testing, patients should be adequately informed about several important aspects (40). First, they should understand the potential advantages, drawbacks, and constraints of the testing process. They should also be aware of the possibility of incidental findings (*i.e.* unexpected genetic variants or mutations unrelated to the primary reason for the genetic analysis) and of the fact that such testing may also have consequences for their family relatives. Concerns have also been raised regarding the privacy and security of genetic data, in particular in the context of commercial direct-to-consumer genomic service (41).

Precision medicine might also raise socioeconomical issues. A first concern is the risk of discrimination in employment or health insurance addressed by the emerging concept of “genetic discrimination” (42). Another point to consider is the cost which might lead to an unequal access to precision medicine (43).

## 6 Conclusion

In conclusion, the integration of genomics is very promising to improve not only SpA daily management, but also to develop new innovative therapies. However, to obtain the full benefits of genomic medicine in SpA, genetic architecture of the disease has to be better understood. There is also a need for strong collaboration between researchers and clinicians. Finally, ethical and social issues related to precision medicine should not be neglected.

## Author contributions

EF: Writing – original draft. MB: Writing – review & editing. FC: Writing – original draft, Writing – review & editing.
